# Nudge-based patient education by pharmacists to promote self-care behaviors for preventing and mitigating chemotherapy-induced skin toxicity: rationale, design, and study protocol of the PHARM-NUDGE trial

**DOI:** 10.1186/s40780-026-00556-4

**Published:** 2026-02-21

**Authors:** Yuka Ito, Koji Suzuki, Masahiro Hatori, Takahiko Yagi, Yasunori Miyamoto, Hikaru Sato, Shuichi Watabe, Naoki Shibata, Yume Otsuka, Tatsuya Yagi, Junichi Kawakami

**Affiliations:** 1 Department of Hospital Pharmacy, Hamamatsu University School of Medicine, Hamamatsu, Japan; 2 Department of Pharmacy, Cancer Institute Hospital, Japanese Foundation for Cancer Research, Tokyo, Japan; 3 Department of Pharmacy, Hamamatsu Medical Center, Hamamatsu, Japan

**Keywords:** Patient education, Nudge, Cancer chemotherapy, Skin toxicity, Randomized controlled trial

## Abstract

**Background:**

Nudge strategies are well-established in behavioral economics as effective approaches for promoting desirable behaviors. However, the potential benefits of integrating nudge-based strategies into pharmacist-led patient education have not yet been demonstrated. Here, we present a study protocol for an interventional trial to address this issue.

**Methods:**

The PHARM-NUDGE study is a multicenter, randomized, parallel-group, single-blind, controlled trial prospectively designed to evaluate whether nudge-based pharmacist-led education can promote patients’ preventive behaviors against skin toxicities associated with cancer chemotherapy. The key inclusion criteria are as follows: (1) patients who are men or women and aged 18 years or older and (2) patients scheduled to receive a chemotherapy regimen containing capecitabine, liposomal doxorubicin, lenvatinib, cetuximab, or panitumumab in outpatient chemotherapy units or during hospitalization. The enrolled patients are randomly assigned in a 2:1 ratio to the nudge-based education or standard education groups. Pharmacists responsible for patient education utilize special educational tools that incorporate nudge strategies and provide skincare education to patients assigned to the nudge-based education group. Patients assigned to the standard education group receive skincare education with equivalent content but without nudges. The primary endpoint is the proportion of patients in each group who achieve four or more of the five predefined behavioral criteria.

**Conclusions:**

The PHARM-NUDGE study is the first randomized controlled trial to evaluate the potential benefits of integrating nudge strategies into pharmacist-led skincare education for patients undergoing cancer chemotherapy with a high risk of skin toxicity, with patient enrollment initiated on October 15, 2025. Completion of the trial and acquisition of the final results are eagerly anticipated.

**Trial registration:**

This trial was registeredwith the Japan Registry of Clinical Trials (clinical trial number: jRCT1040250089, registration date: September 3, 2025).

**Supplementary Information:**

The online version contains supplementary material available at 10.1186/s40780-026-00556-4.

## Background

Skin toxicities, which are often symptomatic and can affect patients’ quality of life, are among the most common adverse events during cancer chemotherapy. Certain types of skin toxicities are strongly associated with specific anticancer agents, including hand–foot syndrome (HFS) induced by fluoropyrimidines and acneiform rash induced by epidermal growth factor receptor inhibitors. Severe skin toxicity may result in dose reduction and treatment discontinuation in patients with cancer [1]. Management and treatment of these skin toxicities have been established and include systemic antibiotics and topical steroids [2]. In addition to these medical interventions, patient self-care practices to minimize skin irritation play an important role in the prevention and mitigation of skin toxicities [3].

Clinical practice guidelines recommend that patients at risk for HFS use skin moisturizers without alcohol, avoid mechanical stress (including long walks or heavy carrying), and avoid chemical stress (including solvents or disinfectants) [4, 5]. In addition, a randomized phase III trial demonstrated that the prophylactic use of 10% urea cream was more effective than non-urea moisturizers in preventing HFS [6]. For patients at risk of acneiform rash induced by epidermal growth factor receptor inhibitors, avoiding frequent hot-water washing and using a sunscreen with high sun protection factor are recommended as part of routine skincare [4, 5]. Although desirable skincare behaviors have been established through previous clinical trials and clinical experience, it should be emphasized that these behaviors are ultimately carried out by patients themselves rather than by healthcare professionals. Pharmacists seek to promote preventive skincare behaviors by educating patients prior to cancer chemotherapy. However, a survey conducted in Japan reported that patient adherence to voluntary skincare behaviors was not necessarily high [7]. Even the behavior with the highest adherence—applying a moisturizer—was performed by only 74.1%, whereas the behavior with the lowest adherence—using sunscreen on exposed areas—was reported by only 14.1% [7].

In behavioral economics, a “nudge” refers to a strategy that alters the choice environment to encourage desirable behaviors without coercion or financial incentives [8]. Nudge-based strategies are increasingly being integrated into clinical settings to promote desirable behaviors among patients and healthcare professionals. A representative example is the use of default options in electronic prescribing systems, in which setting generic drugs as the default has been shown to substantially increase prescription rates for generic drugs compared with brand-name drugs, while preserving clinicians’ discretion [9]. Although the demand for nudge-based approaches in healthcare is increasing, the effect of nudge-based patient education on promoting voluntary skincare behaviors among patients undergoing cancer chemotherapy with a high risk of skin toxicity remains unclear.

The PHARM-NUDGE (PHARMacist-led patient education with NUdges to promote Dermatological self-care during chemotherapy: a General Education-controlled) trial is a multicenter, randomized, parallel-group, single-blind, controlled trial prospectively designed to evaluate the potential benefits of integrating nudge-based strategies into patient education by pharmacists.

## Methods

### Study type, registration, and ethics

The PHARM-NUDGE trial is a multicenter, randomized, parallel-group, single-blind (patient-blinded), controlled trial, with patient enrollment initiated on October 15, 2025. This study is being conducted at Hamamatsu University Hospital (Hamamatsu, Japan), Cancer Institute Hospital of the Japanese Foundation for Cancer Research (Tokyo, Japan), and Hamamatsu Medical Center (Hamamatsu, Japan). This trial was registered with the Japan Registry of Clinical Trials (clinical trial number: jRCT1040250089, registration date: September 3, 2025) and was approved by the Ethics Committee of the Hamamatsu University School of Medicine (approval number: 25–133).

### Patients

The key inclusion criteria are listed in Table 1. Eligible patients must meet all of the following criteria: (1) male or female patients aged 18 years or older; (2) patients scheduled to receive a chemotherapy regimen containing capecitabine, liposomal doxorubicin, lenvatinib, cetuximab, or panitumumab in outpatient chemotherapy units or during hospitalization; and (3) patients who provide written informed consent to participate in this trial. Patients who remain hospitalized between the first and second cycles of chemotherapy are excluded from the study because evaluating changes in voluntary behavior at home would be difficult. The other exclusion criteria are listed in Table 1.

**Table 1:** Key inclusion and exclusion criteria

Key inclusion criteria
1. Male or female patients aged 18 years or older
2. Patients scheduled to receive a chemotherapy regimen containing capecitabine, liposomal doxorubicin, lenvatinib, cetuximab, or panitumumab in outpatient chemotherapy units or during hospitalization
3. Patients who provide written informed consent to participate in this trial
**Key exclusion criteria**
1. Patients with impaired activities of daily living
2. Patients who are unable to communicate effectively
3. Patients for whom the evaluation of this study would be difficult because of underlying disease or comorbidities
4. Patients for whom participation in this study would be difficult because of neurological or psychiatric disorders
5. Patients who remain hospitalized between the first and second cycles of chemotherapy
6. Patients presenting with significant dermatological adverse events at the time of enrollment because of prior treatment
7. Patients with an Eastern Cooperative Oncology Group performance status of two or higher
8. Patients deemed inappropriate for participation by the attending physician or principal investigator

All inclusion criteria must be met for enrollment. Patients meeting any of the exclusion criteria will be excluded

### Study design

**Fig. 1:**
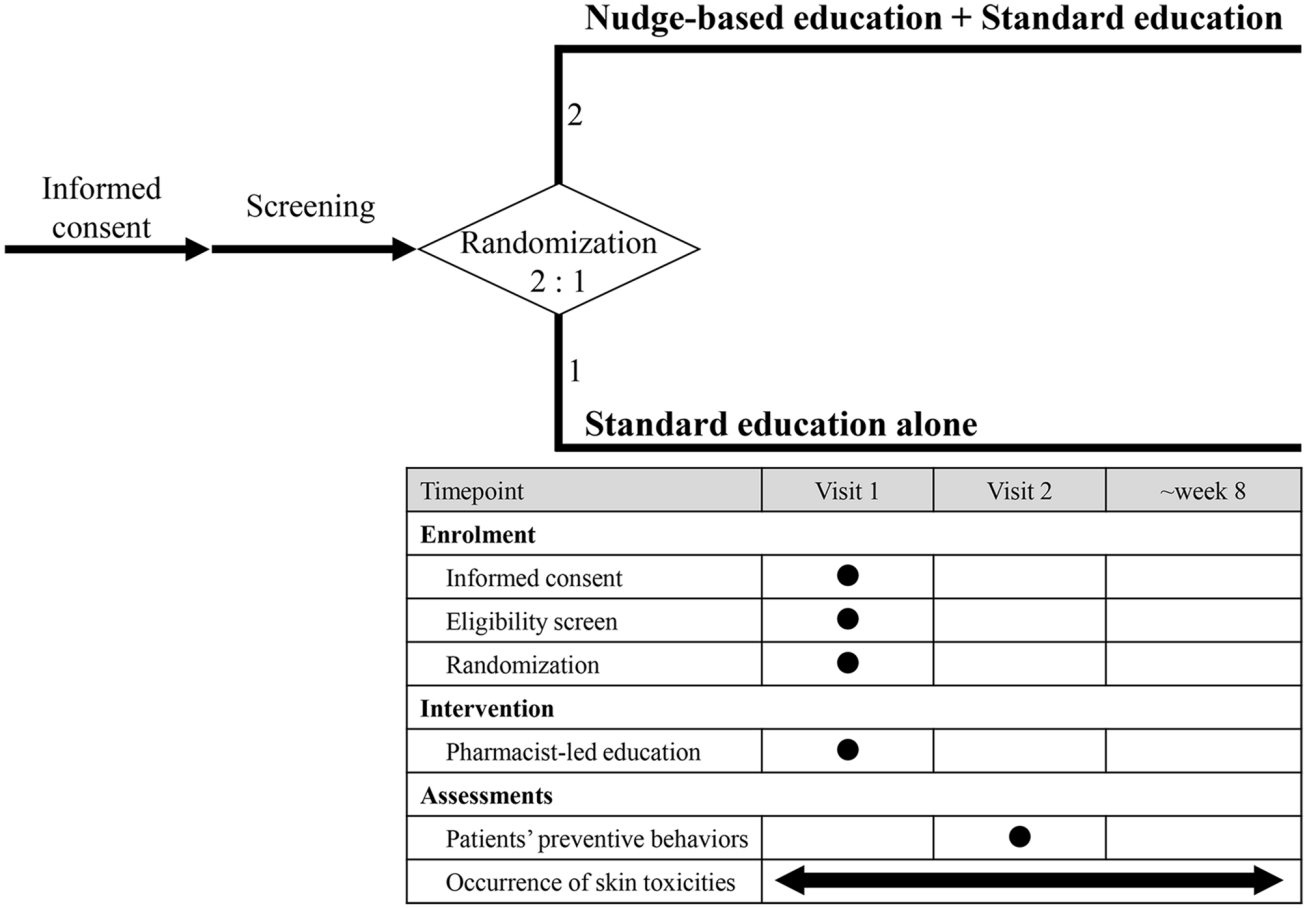
Study design of the PHARM-NUDGE study

An overview of the PHARM-NUDGE study is presented in Fig. 1. The enrolled patients are randomly assigned in a 2:1 ratio to the nudge-based education plus standard education or standard education alone groups. Randomization is performed using block sizes of three and six, which vary randomly, and is stratified according to the cancer chemotherapy regimen administered to each patient. Two study visits are scheduled for each participant: visit 1 on the first day of the chemotherapy regimen and visit 2 on the day of the second administration. At visit 1, each participant receives pharmacist-led education on preventive skincare practices depending on their assigned group (nudge-based education plus standard education or standard education alone). Patients’ self-care behaviors at home are assessed at visit 2. After enrollment, all participants are followed for eight weeks to assess skin toxicity.

### Patient education

**Fig. 2:**
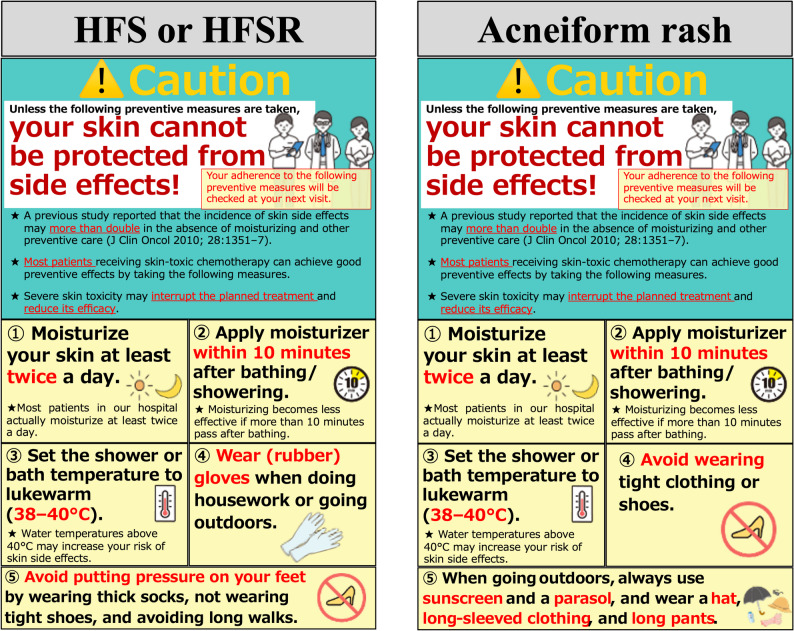
Educational tools that incorporate nudge strategies. Two educational tools were prepared. One tool is designed for patients scheduled to receive chemotherapy regimens containing capecitabine, liposomal doxorubicin, or lenvatinib, which are strongly associated with hand–foot syndrome (HFS) or hand–foot skin reaction (HFSR). The other tool is prepared for patients scheduled to receive chemotherapy regimens containing cetuximab or panitumumab, which are strongly associated with acneiform rash. The Japanese-language version is used in the actual study

The pharmacists responsible for patient education in the PHARM-NUDGE study use special educational tools that incorporate nudge strategies and provide skincare education to patients assigned to the nudge-based education group at visit 1. We prepared two types of educational tools that incorporate several nudges and simplified messages, tailored to the specific features of different skin toxicities (Fig. 2). One tool is designed for patients scheduled to receive chemotherapy regimens containing capecitabine, liposomal doxorubicin, or lenvatinib, which are strongly associated with HFS or hand–foot skin reaction. The other tool is prepared for patients scheduled to receive chemotherapy regimens containing cetuximab or panitumumab, which are strongly associated with acneiform rash. Example sentences from our educational tools, along with their respective nudge types, are presented in Table 2. Patients assigned to the standard education group receive skincare education using the actual tools and explanations used in daily clinical practice at each study institution. In our study design, patients assigned to the nudge-based education group will receive additional explanatory materials on measures to prevent and manage skin toxicities, in addition to general information materials about chemotherapy. To avoid any effects on outcomes from distributing the additional materials themselves, independent of the nudge elements, we also provided patients in the standard education group with supplementary materials on skin toxicities that did not include any nudge elements (Supplementary Fig. S1). Additionally, to minimize differences in educational content and time spent between the two groups, we plan to provide pharmacists responsible for patient education with prior instruction on key points and time allocation.

**Table 2:** Example sentences from our educational tools and their respective nudge types

Nudge type	Sentence
**“Loss aversion”**: People tend to be more sensitive to potential losses or harm than to gains.	“Unless the following preventive measures are taken, your skin cannot be protected from side effects!”
	“A previous study reported that the incidence of skin side effects may more than double in the absence of moisturizing and other preventive care.”
	“Moisturizing becomes less effective if more than 10 minutes pass after bathing.”
	“Water temperatures above 40 °C may increase your risk of skin side effects.”
**“Watching eye effect”**: People tend to change their behavior when they feel they are being observed.	“Your adherence to the following preventive measures will be checked at your next visit.”
**“Social norms”**: People tend to follow what others do.	“Most patients receiving skin-toxic chemotherapy can achieve good preventive effects by taking the following measures.”
	“Most patients in our hospital actually moisturize at least twice a day.”

### Primary endpoint

In the PHARM-NUDGE study, five behavioral criteria are predefined (Table 3). The primary endpoint, defined based on expert consensus among six specialized pharmacists, including Board certified Senior Oncology Pharmacists, is the proportion of patients who meet four or more of the five behavioral criteria in each group. We also estimate 95% confidence intervals (CIs) for the proportions of the two groups.

**Table 3:** Predefined behavioral criteria for evaluating patients’ self-care behaviors

Behavioral criteria for patients scheduled to receive a chemotherapy regimen containing capecitabine, liposomal doxorubicin, or lenvatinib
1. The behavior of moisturizing at least twice a day
2. The behavior of setting the shower or bath temperature to lukewarm (38–40°C)
3. The behavior of applying moisturizers within 10 minutes after bathing
4. The behavior of avoiding irritation of the hands (e.g., by wearing protective [rubber] gloves during housework or outdoor activities)
5. The behavior of avoiding irritation of the feet (e.g., by wearing thick socks and avoiding prolonged walking)
**Behavioral criteria for patients scheduled to receive a chemotherapy regimen containing cetuximab or panitumumab**
1. The behavior of moisturizing at least twice a day
2. The behavior of setting the shower or bath temperature to lukewarm (38–40°C)
3. The behavior of applying moisturizers within 10 minutes after bathing
4. The behavior of avoiding wearing tight-fitting clothing or shoes
5. The behavior of avoiding direct sunlight (ultraviolet exposure) when going outside by using sunscreen, a parasol, a hat, and wearing long-sleeved clothing and long pants

### Secondary endpoint

Five secondary endpoints are predefined: (1) self-implementation rates for each behavioral criterion described in Table 3; (2) the absolute difference between the two groups in the proportion of patients who achieve the primary endpoint; (3) visual analog scale scores for changes in patients’ motivation to perform self-skincare behaviors after pharmacist-led education; (4) incidence rates of dermatological adverse events of grade ≥2 according to the Common Terminology Criteria for Adverse Events 6.0 [10]; and (5) the rate of self-purchase of moisturizers by participants in the absence of a prescription from medical institutions.

### Sample size calculation

In the PHARM-NUDGE study, the target enrollment is a minimum of 120 participants. The sample size is determined by considering 95% CIs for the population proportion of the primary endpoint. The 95% CIs for the population proportion are calculated using the Wald method as follows:

1 $${\hat p \pm 1.96 \times \sqrt {\frac{{\hat p\left( {1 - \hat p} \right)}}{n}} }$$

where *p*^*^*^ is the observed proportion and *n* is the sample size.

Assuming that 70 out of 80 participants achieve the primary endpoint in the nudge-based education group, the implementation rate and 95% CI are 0.88 and 0.80 to 0.95, respectively. In the standard education group, if 20 out of 40 participants achieve the primary endpoint, the implementation rate and 95% CI are 0.50 and 0.35 to 0.65, respectively. Based on these assumptions, the difference in the implementation rates between the two groups is 0.38 (95% CI, 0.20–0.55), which is calculated using the following formula based on the Wald method:

2 $${\left( {{{\hat p}_1} - {{\hat p}_2}} \right) \pm 1.96 \times \sqrt {\frac{{{{\hat p}_1}\left( {1 - {{\hat p}_1}} \right)}}{{{n_1}}} + \frac{{{{\hat p}_2}\left( {1 - {{\hat p}_2}} \right)}}{{{n_2}}}} }$$

where *p*^*^*^_*1*_ and *p*^*^*^_*2*_ denote the observed proportions in the two groups, and *n1* and *n2* denote the respective sample sizes.

### Subgroup analyses

Stratified analyses will be performed by sex, chemotherapy regimen, cancer type, prior treatment status, enrollment institution, and the pharmacist who provides patient education.

### Data collection and management

The five behavioral criteria are assessed at Visit 2 through interviews conducted by pharmacists who have received prior training. Changes in patients’ motivation to perform self-skincare behaviors after pharmacist-led education are evaluated using visual analog scales at Visit 2. Incidence rates of dermatological adverse events are monitored by the attending physicians from Visit 1 to Week 8. The rate of self-purchase of moisturizers by participants is assessed at Visit 2 through interviews conducted by pharmacists. We have a data monitoring committee at Hamamatsu University Hospital (Hamamatsu, Japan) that is independent of the pharmacists responsible for data collection. Data collected at each study site are centralized and managed by the data monitoring committee through an electronic data capture system.

### Statistical analysis

Standardized mean differences will be used for between-group comparisons of patient baseline characteristics. CIs for the proportion of patients achieving the primary endpoint in each group will be calculated using *Formula 1*. CIs for the absolute difference between the two groups in the proportion of patients who achieve the primary endpoint will be calculated using *Formula 2*. Between-group comparisons for continuous variables will be performed using *t*-tests or Mann–Whitney *U* tests. Group comparisons for categorical variables will be conducted using chi-square tests or Fisher’s exact tests. *p* values < 0.05 are considered statistically significant.

## Discussion

The PHARM-NUDGE study is a multicenter, randomized, parallel-group, single-blind, controlled trial designed by pharmacists to evaluate the potential benefits of integrating nudge strategies into patient education. Patient enrollment started on October 15, 2025, and includes patients scheduled to receive cancer chemotherapy regimens that are strongly associated with dermatological adverse events. Although the effect of nudge strategies on promoting desirable behaviors has been demonstrated in various fields, including healthcare, there is no robust evidence on whether nudge-based, pharmacist-led education can promote patients’ preventive behaviors against skin toxicities associated with cancer chemotherapy. To the best of our knowledge, this is the first prospective interventional study with the potential to fill this evidence gap, and the completion of the trial and acquisition of the final results are eagerly anticipated.

Many anticancer drugs have been reported to be associated with skin toxicities. In the PHARM-NUDGE study, we prospectively enroll patients scheduled to receive a chemotherapy regimen containing capecitabine, liposomal doxorubicin, lenvatinib, cetuximab, or panitumumab in outpatient chemotherapy units or during hospitalization. These anticancer drugs are known to cause HFS, hand–foot skin reaction, and acneiform rashes [2]. They are also key components of several chemotherapy regimens routinely administered in outpatient chemotherapy units, including capecitabine plus oxaliplatin and lenvatinib plus pembrolizumab. Based on our preliminary survey of prescription practices at each study site (data not shown), we expected that the majority of the enrolled patients would be outpatients. To minimize the additional time burden on outpatient participants, we planned to provide patient education during intravenous administration in outpatient chemotherapy units. Given these practical considerations, we did not include patients receiving single-agent oral anticancer therapy (including regorafenib, sunitinib, or osimertinib) because it would have been difficult for hospital pharmacists to secure sufficient time for education in cases without infusion therapy in outpatient chemotherapy unit or during hospitalization. Therefore, the five drugs were selected as target agents because they are frequently prescribed at the participating institutions and are commonly used in combination with intravenous anticancer drugs.

In the PHARM-NUDGE study, randomization is performed using the stratified permuted block method with a 2:1 allocation ratio. Stratified randomization is reasonable for small trials in which treatment outcomes may be affected by known clinical factors [11]. In our trial, the chemotherapy regimen administered to each participant was selected as a stratification factor because it is strongly associated with cancer type, sex, age, and the incidence of dermatological adverse events, all of which may create heterogeneity in the environment and influence patients’ behavioral decisions. The allocation ratio was set at 2:1 to ensure that a larger number of participants are assigned to the nudge-based education group to obtain a more precise 95% CI estimate for this group. The PHARM-NUDGE study was designed not primarily to demonstrate the superiority of nudge-based education over standard education, but to accurately estimate the effect size of nudge-based education in the target population.

In this trial, we prepared special educational tools that incorporate nudge-based approaches (Fig. 2 and Table 2). Loss aversion, a fundamental concept in behavioral economics, refers to the tendency for people to be more sensitive to potential losses than to equivalent gains [12]. A representative example of loss aversion is vaccination promotion. Wang et al. showed that loss-framed messaging significantly increases COVID-19 vaccine acceptance [13]. The watching eye effect is a psychological phenomenon whereby people are more likely to engage in prosocial behaviors when they feel that others are watching them. Gaube et al. showed that visual eye cues improved hand hygiene compliance among healthcare workers [14]. In the PHARM-NUDGE study, although visual eye cues are not used, the educational tools clearly state that patients’ self-care behaviors at home would be checked at their next visit, thereby creating a psychological sense of being observed by healthcare workers. Social norms refer to individuals’ tendency to follow behaviors that are common or expected within a group. For example, telling adolescents with epilepsy how well their peers took their medications increased adherence [15]. Loss aversion, watching eye effects, and social norms have been established as nudge-based approaches, and their effectiveness is anticipated in the PHARM-NUDGE study.

The primary endpoint of the PHARM-NUDGE study was defined according to the behavioral criteria for assessing patients’ self-care behaviors (Table 3). Using moisturizers and avoiding skin irritation are recommended as basic self-care measures in clinical practice guidelines [4, 5]. Therefore, it is important to encourage patients to engage in these behaviors. However, evidence demonstrating that these behaviors can prevent skin toxicity remains inconclusive. A randomized phase III study evaluated the effect of a structured teaching model incorporating intensive prophylactic measures (including routine use of emollients three to four times a day and wearing loose-fitting clothes and shoes) on the prevention of capecitabine-induced HFS; however, the intervention did not demonstrate a statistically significant difference [16]. Although our trial included the incidence of dermatological adverse events as a secondary endpoint, it may be challenging to detect a statistically significant difference between the two groups.

In the PHARM-NUDGE trial, our primary objective is to estimate the incremental effect of integrating nudge-based strategies into standard patient education. To ensure standardization of baseline educational practices, responsibility for patient education was assigned to a single healthcare professional. Nevertheless, we believe that this nudge-based approach is not inherently limited to pharmacists and may be adaptable to other healthcare professionals in future implementations.

Our study protocol has potential limitations. Firstly, differences in the educational materials or in pharmacists’ explanations may allow participants to infer their assigned group. To address this issue, we plan to inform participants only that two types of educational approaches are being compared, without disclosing that a special educational tool will be provided exclusively to one group. Secondly, although we described the study design as a single-blind trial in the Methods section, it is not possible to fully blind the differences in the educational materials between the two groups. Therefore, it should be noted that the trial is not fully single-blinded.

## Conclusions

The PHARM-NUDGE study is the first prospective randomized controlled trial to evaluate the potential benefits of integrating nudge strategies into pharmacist-led skincare education for patients undergoing cancer chemotherapy with a high risk of skin toxicity. Nudge strategies are well-established in behavioral economics as effective approaches for promoting desirable behaviors. The application of nudge strategies to pharmacist-led patient education is anticipated to support the implementation of more effective, evidence-based educational interventions.

## Electronic supplementary material

Below is the link to the electronic supplementary material.

Supplementary material 1

## Abbreviations

HFS: hand–foot syndrome
CI: confidence interval
