# Supplementary material for: Nudge-based patient education by pharmacists to promote self-care behaviors for preventing and mitigating chemotherapy-induced skin toxicity: rationale, design, and study protocol of the PHARM-NUDGE trial
Source: J Pharm Health Care Sci. 2026 Feb 21;12:38. doi: 10.1186/s40780-026-00556-4 (PMC13032257; doi:10.1186/s40780-026-00556-4)
Supplement: Supplementary file 1 — Supplementary material 1 [file 40780_2026_556_MOESM1_ESM.pptx]

## Slide 1
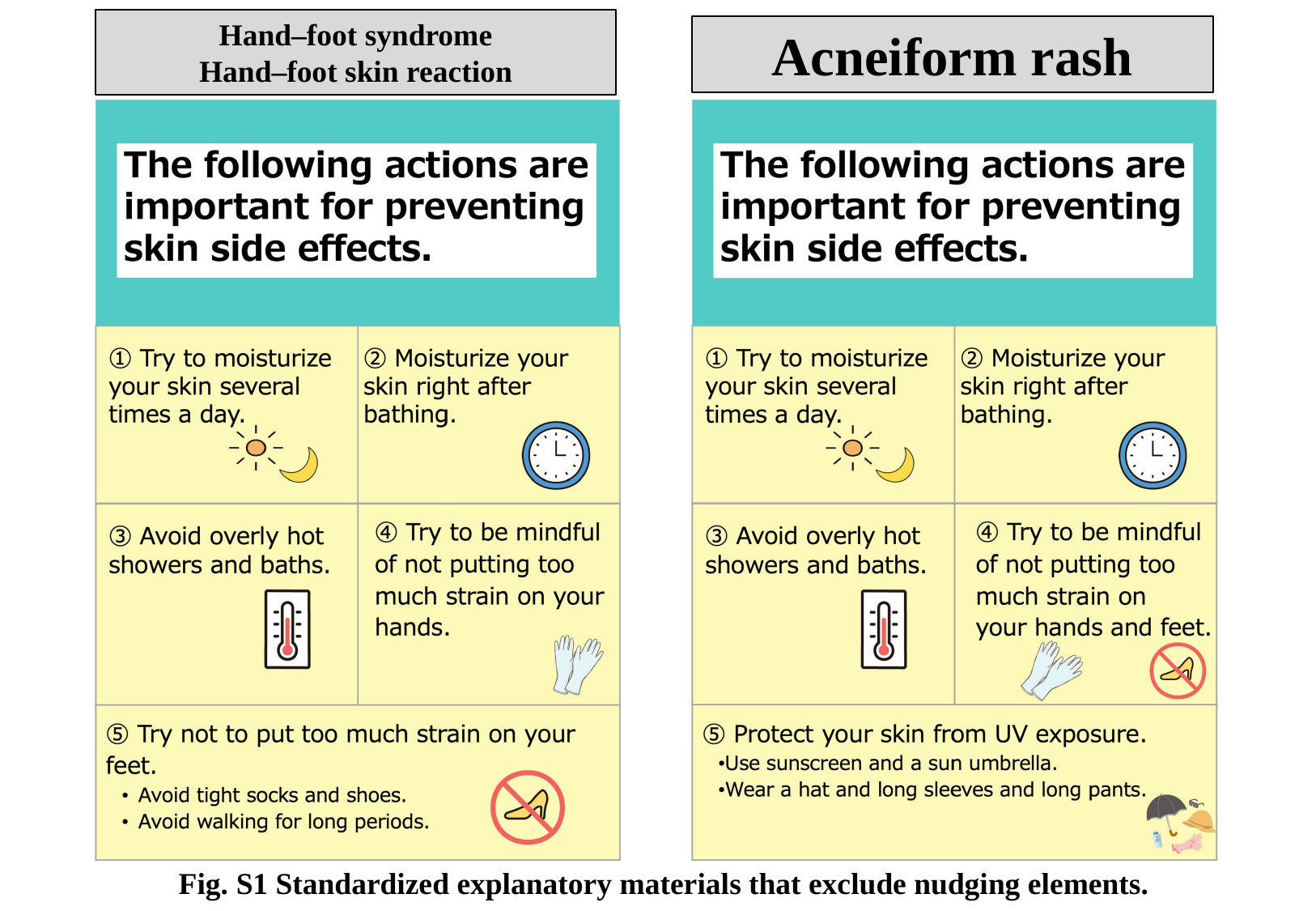

Hand–foot syndrome
Hand–foot skin reaction
Acneiform rash
Fig. S1 Standardized explanatory materials that exclude nudging elements.
